# The antimicrobial effects of zinc oxide-calcium hydroxide mixture fillers: determining the ideal mixture ratio

**Published:** 2019-06

**Authors:** Ali Nozari, Ali Karimkhani, Mohammad Motamedifar, Payam Arasteh

**Affiliations:** 1 Department of Pediatric Dentistry, School of Dentistry, Shiraz University of Medical Sciences, Shiraz, Iran; 2 Department of Bacteriology and Virology, Shiraz HIV/AIDS Research Center, Institute of Health, Shiraz School of Medicine, Shiraz University of Medical Sciences, Shiraz, Iran; 3 Department of Foreign Languages and Linguistics, Shiraz University, Shiraz, Iran

**Keywords:** Zinc oxide, Calcium hydroxide, Antimicrobial, Primary teeth, Root canal filling material

## Abstract

**Background and Objectives:**

Although zinc oxide (ZO)-calcium hydroxide (CaOH) mixtures have been successful regarding their absorption rate compatibility with dissolving primary teeth, no study has been conducted on the appropriate mixture ratio to obtain effective antibacterial properties. In this study, we compared antibacterial activity of CaOH-ZO pastes using different mixture ratios sagainst *Enterococcus faecalis* as an important bacterium in root canal treatment failure.

**Materials and Methods:**

Seven types of pastes were prepared in our laboratory. The first group included one gram of ZO+eugenol, second group included one gram of CaOH+distilled water, third group included 0.5gram ZO+0.5gram CaOH+distilled water (1:1), forth group included 0.75gramCaOH+0.25gramZO+distilled water (3:1), the fifth group included 0.33gram of CaOH+0.66gram of ZO+distilled water (1:2), the sixth group included 0.75gram of ZO+0.25 CaOH+distilled water (3:1), the seventh group included 0.66 gram CaOH+0.33 gram ZO+distilled water (2:1), and the final group included one gram of gelatin+distilled water (as the control group). These pastes were compared regarding their antibacterial effects against *Enterococcus faecalis* using agar diffusion and microdilution methods.

**Results:**

Except for the control group, all prepared pastes showed antibacterial properties. Order of minimum inhibitory concentration for pastes were as followed: CaOH-ZO (1:3)=CaOH-ZO (1:2)>CaOH-ZO (1:1)>CaOH-ZO (3:1)=CaOH-ZO (2:1)>CaOH=ZO-eugenol. Order of minimum bactericidal concentration, which shows a weaker bactericidal effect, according to type of paste, were as followed: CaOH-ZO (1:3)>CaOH-ZO mixture (1:2)>CaOH-ZO mixture (1:1)>CaOH-ZO mixture (3:1)=CaOH-ZO (2:1)>CaOH=ZO-eugenol. Only CaOH-ZO (1:3) and CaOH-ZO (1:2), showed significantly weaker MICs and MBCs (p < 0.001).

**Conclusion:**

Considering the limitations of an *in-vitro* study, in terms of anti-bacterial effects against *Enterococcus faecalis*, CaOH-ZO mixture (2:1) is equivalent to ZO-eugenol as the most commonly used material in polypectomy of primary teeth.

## INTRODUCTION

Dental caries and trauma are two main causes of primary teeth pulp involvement. To date, many advancements have been achieved regarding the prevention of dental caries and the importance of preserving the teeth has attracted much attention (1, 2).

Infections related to the root canal of the primary teeth have a polymicrobial nature and anaerobic bacteria are considered to be the dominant species involved in this process. Among these, *Enterococcus faecalis*, is considered the most important and the most resistant bacteria in the canal. These microorganisms are released along the root canal and may remain in the canals even after biomechanical preparation of canals and the use of detergents (3).

Root canal fillers are used to remove residual pathogens and to neutralize their toxic products and prevent re-infection of the canals (4, 5). A paste consisting of zinc oxide eugenol, iodoform and calcium hydroxide are the three main categories of materials used to fill the root canal of primary teeth. Among these fillers, zinc oxide eugenol, is the most common substance used to fill the root canal of primary teeth (6).

Due to the small antibacterial effects of zinc oxide eugenol and its slower adsorption rate in comparison to the roots of primary teeth, remnants exited between the root zone and apex of the tooth after exfoliation of primary teeth, causes the deformation of the underlying permanent tooth (7). Moreover, foreign body reactions and cytotoxic effects have also been reported with this type of filler (8). Therefore, the ideality of this substance as a canal filler in primary teeth has been a subject of controversy.

The combination of iodoform and calcium hydroxide, which are commercially available as Vitapoxy or Metapex, are absorbed rapidly from the apex and intracanal regions and this absorption is faster than the physiological dissolving of the primary tooth. On the other hand, iodoform material may produce an allergic reaction (9, 10).

The other group of dental canal fillers for primary teeth is calcium hydroxide based materials. Despite their excellent biological properties (11), these materials are not good for clinical use due to their tissue fluid permeability, radiolucency, higher absorption rate than dissolving primary teeth (12).

In order to overcome the mentioned disadvantages, the combination of zinc oxide (without eugenol) with calcium hydroxide (zinc oxide-calcium hydroxide mixture) has been studied. These combinations are meant to reduce phagocytosis and to achieve the same absorption rate with that of the dissolving primary teeth by modifying the physical and chemical characteristics of the paste (11, 13). Although these pastes have been successful regarding their absorption rate compatibility with the dissolving primary teeth, no study has been conducted on the appropriate mixture ratio to obtain appropriate antibacterial properties.

In this study we aimed to evaluate and compare the antibacterial activity of calcium hydroxide-zinc oxide mixtures against *E. faecalis* using different mixture ratios.

## MATERIALS AND METHODS

### Study protocol and design.

This experimental *in-vitro* study was conducted in Shiraz Dental School, affiliated to Shiraz University of Medical Sciences, Shiraz, Iran. Seven types of pastes were prepared in the laboratory of Shiraz Dental School, Shiraz, Iran. All pastes were formed by a mixture of a specific powder and a solvent. Powders included zinc oxide (Kemdent, Swindon, England), calcium hydroxide (Dentonics, Master-Dent, Carolina, USA) and gelatin (Rousselot, Brasillia, Brazil), (as control) and solvents included distilled water and eugenol (Kemdent, Swindon, England).

The combination used to form each paste mixture was as followed: the first group included one gram (g) of zinc oxide + eugenol, second group included one g of calcium hydroxide powder + distilled water, third group included 0.5g zinc oxide powder + 0.5g calcium hydroxide powder + distilled water (1:1), forth group included 0.75g calcium hydroxide + 0.25g zinc oxide + distilled water (3:1), the fifth group included 0.33g of calcium hydroxide + 0.66g of zinc oxide + distilled water (1:2), the sixth group included 0.75g of zinc oxide + 0.25 calcium hydroxide + distilled water (3:1), the seventh group included 0.66g calcium hydroxide + 0.33g zinc oxide + distilled water (2:1), and the final group included one g of gelatin + distilled water (as the control group).

For the evaluation of antibacterial effects of the pastes, the agar diffusion inhibitory test using the pour plate method and the microdilution test were used to evaluate Minimum Bactericidal Concentration (MBC) and Minimum Inhibitory Concentration (MIC). Antimicrobial tests were conducted in the Microbiology Department of Shiraz University of Medical Sciences, Shiraz, Iran.

### Agar diffusion tests.

For the agar diffusion inhibitory test using the Pour Plate method, a standard strain of *E. faecalis* ATCC1700 (American Type Culture Collection) was checked. The strain was inoculated into a liquid environment (Brain Heart Infusion broth, Merck, Darmstadt, Germany) and was then incubated for 48 hours at 37°C. The sample then was transferred onto a solid environment (Triptic Soy Agar, Merck, Darmstadt, Germany) and was then cultured for 48 hours to obtain bacterial colonies. Using the obtained colonies and using normal saline, a suspension with a concentration equal to 0.5 McFarland turbidity standard (almost containing 1.5 ×10^8^ cells/ml) was prepared. Concentration of the suspensions was assessed and confirmed using spectrophotometry (Sartoriuste, Gelatin, Germany) at a wavelength of 600 nm. After autoclaving the Muller Hinton Agar medium (Merck, Darmstadt, Germany) it cooled down at 45–50°C and subsequently 20 mL was drawn out and added to the plates with one mL of bacteria with a concentration equal to 0.5 Mcfarland. The agar thickness reached 4 mm, it was allowed to cool down. After that, in each plate, wells with a diameter of 4 mm × 6 mm were created and one g of the prepared pastes was added to each well. The plates were then incubated at 37°C for 24 hours. Using a caliper, the minimum distance between the outer part of the wells and the first point at which microorganisms showed growth, was measured. Negative and positive control wells were included (14). For each paste this measurement was repeated five times and mean of these measurements were recorded.

### Microdilution method for determining MBC and MIC.

For the microdilution method, 96-well plates were utilized. One gram of the combinations was initially mixed with 1.5 mL of solvent (eugenol or water) and the final obtained volume of two mL was used. From this mixture, 100 μl was extracted. For the dilution process, 100 μl of the mixture was used and inoculated into the first and second of horizontal wells, after which 100 ul of Muller Hinton Broth was inoculated into the second well. Following which, another 100 ul was extracted from the second well and inoculated into the third well and 100 μl of the broth was added to the well. This process was continued until the tenth well. For the next stage 90 ul of the Muller Hinton Broth plus 10 μl of bacterial suspension with a concentration equal to 0.5 Mcfarland was added to each of the horizontal wells. The concentrations ranged from 1/2 -1/1024.

Each of these plates, which included different concentrations of the paste, was incubated at 37°C for 24 hours. Then, 10 μl from each well was drawn out and one drop was inoculated onto a blood agar plate and was then spun using an “L” shaped tube so that the liquid-containing bacteria would spread on the growth plate. After 24 hours, the plate were investigated for bacterial growth. Assessment of bacterial growth was done by counting number of bacterial colonies. MIC, was considered as the lowest concentration of paste that did not show bacterial growth, MBC, was considered as the lowest concentration of paste at which the colony count was less than 0.1% of original inoculum on a single spot (14, 15). The micro-dilution series was repeated three times for each paste and at each repetition the MIC and MBC was recorded and mean values was calculated.

### Statistical analysis.

The Statistical Package for Social Sciences software (SPSS Inc., Chicago, IL, USA), version 20 for windows, was used for analysis of data. For comparison of quantitative data between the groups the one-way ANOVA test was used and for between group comparisons the post hoc Duncan test was utilized. Data are presented as mean and standard deviations (SD) and maximum and minimum. A p-value of less than 0.05 was considered statistically significant.

## RESULTS

The agar diffusion inhibitory test showed that, except for the control group, all the prepared pastes had antibacterial properties (Table 1).

**Table 1.** Results of the agar diffusion inhibitory test.^*^

**Variables**	**Mean (SD) (mm)**	**Minimum**	**Maximum**
Zinc oxide-Eugenol‡	11.80 ± 0.447	11	12
Calcium hydroxide	19 ± 0.707	18	20
Calcium hydroxide-zinc oxide (1:1)	19.80 ± 1.095	18	21
Calcium hydroxide-zinc oxide (3:1)	20 ± 1.225	18	21
Calcium hydroxide-zinc oxide (1:3)	17.60 ± 2.302	14	20
Calcium hydroxide-zinc oxide mixture (1:2)	17.20 ± 1.483	15	19
Calcium hydroxide-zinc oxide mixture (2:1)	18 ± 1.225	16	19
Eugenol†	12 ± 0.466	11	13
Gelatin (control)	-	-	-

* Each test was repeated five consecutive times.

† The pure eugenol paste was also tested in the agar diffusion inhibitory test.

‡ The first group includes one gram(g) of zinc oxide + eugenol, second group includes one g of calcium hydroxide powder+distilled water, third group includes 0.5g zinc oxide powder+0.5g calcium hydroxide powder+distilled water (1:1), forth group includes 0.75g calcium hydroxide+0.25g zinc oxide + distilled water (3:1), the fifth group includes0.33g of calcium hydroxide+0.66g of zinc oxide+distilled water (1:2), the sixth group includes 0.75g of zinc oxide+0.25 calcium hydroxide+distilled water (3:1), the seventh group includes 0.66g calcium hydroxide+0.33g zinc oxide+distilled water (2:1), and the final group includes one g of gelatin+distilled water (control group).

Results of the micro-dilution tests showed that mean (SD) MIC of zinc oxide-eugenol paste and calcium hydroxide were similar (0.0045 ± 0.0030). Moreover, MIC of calcium hydroxide-zinc oxide (1–3) and calcium hydroxide-zinc oxide mixtures (1:2) were similar (0.0260 ± 0.0090 mg/μl) and statistically higher than other prepared pastes (p < 0.001), which shows a weaker antibacterial effect. Order of MIC for the pastes were as followed: calcium hydroxide-zinc oxide mixture (1:3) = calcium hydroxide-zinc oxide mixture (1:2) > calcium hydroxide-zinc oxide mixture (1:1) > calcium hydroxide-zinc oxide mixture (3:1) = calcium hydroxide-zinc oxide mixture (2:1) > calcium hydroxide= zinc oxide eugenol (Table 2).

**Table 2.** Minimum inhibitory concentration among different paste combinations.^*^

**Variables†**	**Mean (SD)**	**Minimum**	**Maximum**
Zinc oxide-Eugenol- ug/μl	4.5 ± 3^a^	1.9	7.8
Calcium hydroxide - ug/μl	4.5 ± 3^a^	1.9	7.8
Calcium hydroxide-zinc oxide (1-1) - ug/μl	10.4 ± 4.5^a^	7.8	15.6
Calcium hydroxide-zinc oxide (3-1) - ug/μl	5.2 ± 2.2^a^	3.9	7.8
Calcium hydroxide-zinc oxide (1-3) - ug/μl	26 ± 9^b^	15.6	31.2
Calcium hydroxide-zinc oxide mixture (1:2) - ug/μl	26 ± 9^b^	15.6	31.2
Calcium hydroxide-zinc oxide mixture (2:1) - ug/μl	5.2 ± 2.2^a^	3.9	7.8

* Each test was repeated three consecutive times.

† Cross comparison of minimum inhibitory concentration between pastes showed that calcium hydroxide-zinc oxide mixture (1:3) and calcium hydroxide-zinc oxide mixture (1:2) had a significantly higher minimum inhibitory concentration compared to all other pastes (p < 0.001). Superscript alphabets show a significant difference between that of the mentioned pastes.

Regarding MBC, zinc oxide-eugenol paste and calcium hydroxide were similar (0.0065 ± 0.0022 mg/μl). Order of MBCs, which shows a weaker bactericidal effect, according to type of paste were as followed: calcium hydroxide-zinc oxide mixture (1:3) > calcium hydroxide-zinc oxide mixture (1:2) > calcium hydroxide-zinc oxide mixture (1:1) > calcium hydroxide-zinc oxide mixture (3:1) = calcium hydroxide-zinc oxide mixture (2:1) > calcium hydroxide = zinc oxide eugenol.

Cross comparison of MBC between pastes showed that calcium hydroxide-zinc oxide mixture (1:3) and calcium hydroxide-zinc oxide mixture (1:2) had significantly higher concentration of MBC compared to all other mixtures (p < 0.001) (Table 3).

**Table 3.** Minimum bactericidal concentration among different paste combinations.*

**Variables†**	**Mean (SD)**	**Minimum**	**Maximum**
Zinc oxide-Eugenol - ug/μl	6.5 ± 2.2^a^	3.9	7.8
Calcium hydroxide - ug/μl	6.5 ± 2.2^a^	3.9	7.8
Calcium hydroxide-zinc oxide (1-1) - ug/μl	13 ± 4.5^a^	7.8	15.6
Calcium hydroxide-zinc oxide (3-1) - ug/μl	10.4 ± 4.5^a^	7.8	15.6
Calcium hydroxide-zinc oxide (1-3) - ug/μl	31.2 ± 0^b^	31.2	31.2
Calcium hydroxide-zinc oxide mixture (1:2) - ug/μl	41.6 ± 18^b^	31.2	62.5
Calcium hydroxide-zinc oxide mixture (2:1) - ug/μl	10.4 ± 4.5^a^	7.8	15.6

* Each test was repeated three consecutive times.

† Cross comparison of minimum inhibitory concentration between pastes showed that calcium hydroxide-zinc oxide mixture (1:3) and calcium hydroxide-zinc oxide mixture (1:2) had a significantly higher minimum bactericidal concentration compared to all other pastes (p < 0.001). Superscript alphabets show a significant difference between that of the mentioned pastes.

## DISCUSSION

To the best of authors’ knowledge, to date, no study has been conducted on the ideal ratio of calcium hydroxide-zinc oxide mixtures regarding antibacterial properties. In the current study, considering the limitations of the diffusion inhibitory test, including the inability of diagnosing inhibitory or bactericidal effects of materials and the need for controlling of multiple factors to obtain precise test results (16), this test was only used for primary screening. For confirming the antibacterial effects of pastes and for exact evaluation of antibacterial properties, the MIC and MBC were determined.

Results of the study showed that based on the microdilution method, stronger antibacterial properties pertain to zinc oxide-eugenol and calcium hydroxide pastes. The mentioned pastes descriptively have similar antibacterial properties. Furthermore, the only pastes that demonstrated significant difference, were the zinc oxide-calcium hydroxide 2 to 1 ratio and the zinc oxide calcium hydroxide 3 to 1 ratio, which showed higher MIC and MBC (weaker antibacterial effect) compared to other pastes.

In 2012, Sapna et al. studied the antibacterial properties of calcium hydroxide-zinc oxide-distilled water mixture and calcium hydroxide-zinc oxide-sodium fluoride mixture against eight microorganisms that were commonly found in the canal, by means of agar diffusion test. The antibacterial properties of these paste mixtures were compared with those of zinc oxide eugenol, calcium hydroxide (apexical) and andoflas. Their results showed that zinc oxide-eugenol possesses the strongest antibacterial properties as followed: zinc-oxide eugenol > calcium hydroxide-zinc oxide-sodium florid mixture > calcium hydroxide-zinc oxide-distilled water mixture >apexical>metapex (17).

In the mentioned study, the paste mixture made from zinc oxide and calcium hydroxide did not show antibacterial properties against *Enterococcus faecalis* and calcium hydroxide-zinc oxide-sodium florid mixture showed weak antibacterial properties against *Enterococcus faecalis*. This could have been due to culture medium and the limitations of the agar diffusion test, in which calcium hydroxide is prevented from spreading and so it precipitates in agar environment due to high pH levels (17, 18).

The antibacterial properties of zinc oxide eugenol, based on previous studies, has been attributed to the eugenol (18, 19). Our findings also support this, as the agar diffusion method showed the antibacterial properties of pure eugenol to be similar to that of zinc oxide-eugenol. The antibacterial properties of calcium hydroxide has been attributed to its alkaline properties and its free hydroxylion (20, 21).

In our study, pure calcium hydroxide and zinc oxide eugenol had better antibacterial properties in comparison to that of calcium hydroxide-zinc oxide mixtures. This could be attributed to the lack of eugenol in the calcium hydroxide-zinc oxide mixtures which were used, considering that the eugenol in the zinc oxide eugenol carries the actual antibacterial properties (18, 19). Not all studies were in complete coherence with our results.

Reddy et al. in a study, investigated the antibacterial properties of zinc oxide-eugenol, calcium hydroxide-distilled water, zinc oxide +camphor phenol, metapex and zinc oxide-eugenol + formocresol pastes against anaerobic, Gram-positive and Gram-negative bacteria. In their study, in which they also used the agar diffusion method, unlike the results of our study, zinc oxide-eugenol and calcium hydroxide did not show any antibacterial properties against *Enterococcus faecalis*. This difference could be attributed to the limitations of the agar diffusion test and the higher precision that the microdilution test provides. As mentioned previously, due to culture medium and the limitations of agar diffusion test in which calcium hydroxide is prevented from spreading, the performance of this test is limited (17, 18).

In our study, we found that both zinc oxide eugenol and calcium hydroxide pastes possess appropriate antimicrobial properties against *Enterococcus faecalis* and both pastes show similar antibacterial properties. In the calcium hydroxide-zinc oxide mixtures, when the ratio of zinc oxide increased, the antibacterial properties decrease and from a ratio of 2 to 1 (zinc oxide to calcium hydroxide), this decreasing in antibacterial activity becomes statistically significant(p < 0.001). This can be attributed to the fact that this paste lacks eugenol, as studies have attributed the antibacterial effects of zinc oxide-eugenol to the eugenol component (18, 19), and on the other hand by decreasing the calcium hydroxide component the free hydroxide ion, which has the antibacterial effects decreases.

We found that in the calcium hydroxide-zinc oxide mixture, by increasing the ratio of calcium hydroxide to zinc oxide up to 2 to 1, the antibacterial properties increased, however after this increase the antibacterial properties stabilizes. According to the study by Segato et al. (22), a ratio of 2:1 of the calcium hydroxide-zinc oxide paste has the highest alkaline characteristic and the most appropriate physicochemical properties and this characteristic (alkaline) is among the most important determinants of antibacterial effects (20, 21). One can conclude that at this ratio the highest alkaline property is obtained and higher ratios do not render higher alkaline properties, thus showing similar antibacterial effects.

This study was not without limitation. This was an *in-vitro* study, and although this type of study does have some strong points such as a quantitative assessment of antimicrobial effects, it cannot completely simulate the conditions of an *in-vivo* study. Therefore, the results of laboratory based research should only be interpreted according to the conditions and results of clinical studies.

In this study, antibacterial property of pastes were assessed only against *Enterococcus faecalis*, which is considered as the most important and the most resistant bacteria in the canal, and other microorganisms may still exist after pulpectomy treatment. Therefore, further research is needed on other aerobic or anaerobic, Gram positive and negative microorganisms within the canals. We also recommend the use of several different methods in future studies to measure the diameter of the zones of bacterial inhibition in several intervals, moreover studies can also use different substances in the calcium hydroxide-zinc oxide mixture (instead of distilled water) such as sodium fluoride and aloevera with different ratios.

In conclusion, considering the calcium hydroxide-zinc oxide mixture (2:1) is equivalent to zinc oxide eugenol as the most commonly used material in pulpectomy of primary teeth.

## References

[1] Bertassoni LE Orgel JP Antipova O Swain MV The dentin organic matrix - limitations of restorative dentistry hidden on the nanometer scale. Acta Biomater 2012;8:2419–2433. 22414619 10.1016/j.actbio.2012.02.022 PMC3473357

[2] Albino J Tiwari T Preventing childhood caries: a review of recent behavioral research. J Dent Res 2016;95: 35–42. 26438210 10.1177/0022034515609034 PMC4700662

[3] de Paz LEC Aetiology of Persistent Endodontic Infections in Root-Filled Teeth. Apical Periodontitis in Root-Filled Teeth: Springer; 2018 p. 21–32.

[4] Bociong K Szczesio A Krasowski M Sokolowski J The influence of filler amount on selected properties of new experimental resin dental composite. Open Chem 2018;16:905–911.

[5] Matinlinna JP Lung CYK Tsoi JKH Silane adhesion mechanism in dental applications and surface treatments: A review. Dent Mater 2018;34:13–28. 28969848 10.1016/j.dental.2017.09.002

[6] Praveen P Anantharaj A Venkataraghavan K Rani P Sudhir R Jaya A A review of obturating materials for primary teeth. SRM J Res Dent Sci 2011;2:42–44.

[7] Pilownic KJ Gomes APN Wang ZJ Almeida LHS Romano AR Shen Y Physicochemical and biological evaluation of endodontic filling materials for primary teeth. Braz Dent J 2017; 28:578–586. 29215682 10.1590/0103-6440201701573

[8] Jung S Sielker S Hanisch MR Libricht V Schäfer E Dammaschke T Cytotoxic effects of four different root canal sealers on human osteoblasts. PLoS One 2018;13(3):e0194467. 29579090 10.1371/journal.pone.0194467 PMC5868789

[9] Garg N Garg A Kang R Mann J Manchanda SK Ahuja B A comparison of Apical Seal produced by Zinc Oxide Eugenol, Metapex, Ketac Endo and AH plus root canal Sealers. Endodontology 2014;26:252–258.

[10] Jindal P Khurana NS Juneja R Sharma H Kumar V Atypical response of Gingival tissue to extruded Metapex. J Clin Diagn Res 2016;10:ZJ09–ZJ10. 10.7860/JCDR/2016/18571.8014 PMC4963797 27504437

[11] Queiroz AMd Assed S Consolaro A Nelson-Filho P Leonardo MR Silva RAB Subcutaneous connective tissue response to primary root canal filling materials. Braz Dent J 2011;22:203–211. 21915517 10.1590/s0103-64402011000300005

[12] Queiroz AMd Nelson Filho P Silva LABd Assed S Silva RABd Ito IY Antibacterial activity of root canal filling materials for primary teeth: zinc oxide and eugenol cement, Calen paste thickened with zinc oxide, Sealapex and EndoREZ. Braz Dent J 2009;20:290–296. 20069251 10.1590/s0103-64402009000400005

[13] Pinto DN de Sousa DL Araújo RBR Moreira-Neto JJS Eighteen-month clinical and radiographic evaluation of two root canal-filling materials in primary teeth with pulp necrosis secondary to trauma. Dent Traumatol 2011;27:221–224. 21342437 10.1111/j.1600-9657.2011.00978.x

[14] Mahon CR. Lehman DC. Manuselis G (2007). Textbook of Diagnostic Microbiology. 3’rd Edition Saunders USA pp. 319–378.

[15] Andrews JM Determination of minimum inhibitory concentrations. J Antimicrob Chemother 2001;48:S5–16. 10.1093/jac/48.suppl_1.5 11420333

[16] Al-Shwaimi E Evaluation of antimicrobial effect of root canal sealers. Pak Oral Dental J 2011;31:2.

[17] Sapna H Priti Kamlesh L Dinesh Rao B Shubha A An *in vitro* evaluation of antimicrobial efficacy of primary root canal filling materials. J Clin Pediatr Dent 2012;37:59–64. 23342568 10.17796/jcpd.37.1.v6305588xw525505

[18] Reddy S Ramakrishna Y Evaluation of antimicrobial efficacy of various root canal filling materials used in primary teeth: a microbiological study. J Clin Pediatr Dent 2007;31:193–198. 17550046 10.17796/jcpd.31.3.t73r4061424j2578

[19] Almaroof A Niazi S Rojo L Mannocci F Deb S Influence of a polymerizable eugenol derivative on the antibacterial activity and wettability of a resin composite for intracanal post cementation and core build-up restoration. Dent Mater 2016;32:929–939. 27130610 10.1016/j.dental.2016.04.001

[20] Desai S Chandler N Calcium hydroxide–based root canal sealers: a review. J Endod 2009;35:475–480. 19345790 10.1016/j.joen.2008.11.026

[21] Barja-Fidalgo F Moutinho-Ribeiro M Oliveira MAA Oliveira BHd A systematic review of root canal filling materials for deciduous teeth: is there an alternative for zinc oxide-eugenol? ISRN Dent 2011;2011: 367318. 21991471 10.5402/2011/367318 PMC3169841

[22] Segato RAB Pucinelli CM Ferreira DCA Daldegan ADR Silva RSd Nelson-Filho P Physicochemical properties of root canal filling materials for primary teeth. Braz Dent J 2016;27:196–201. 27058384 10.1590/0103-6440201600206

